# The NIAID Discovery Portal: a unified search engine for infectious and immune-mediated disease datasets

**DOI:** 10.1128/msystems.01270-25

**Published:** 2025-12-31

**Authors:** Ginger Tsueng, Emily Bullen, Candice Czech, Dylan Welzel, Leandro Collares, Jason Lin, Everaldo Rodolpho, Zubair Qazi, Nichollette Acosta, Lisa M. Mayer, Sudha Venkatachari, Zorana Mitrović Vučičević, Poromendro N. Burman, Deepti Jain, Jack DiGiovanna, Maria Giovanni, Asiyah Lin, Wilbert Van Panhuis, Laura D. Hughes, Andrew I. Su, Chunlei Wu

**Affiliations:** 1 The Scripps Research Institute 4356, La Jolla, California, USA; 2 Office of Data Science and Emerging Technologies, National Institute of Allergy and Infectious Diseases 35037, Rockville, Maryland, USA; 3 National Cancer Institute, Rockville, Maryland, USA; 4 Velsera, Charlestown, Maryland, USA; 5 National Institute of Allergy and Infectious Diseases 35037, Rockville, Maryland, USA; Qingdao University, Qingdao, Shandong, China

**Keywords:** data reuse, metadata harmonization, infectious disease, immunology, FAIR data, data discovery, resource report

## Abstract

The National Institute of Allergy and Infectious Diseases (NIAID) Data Ecosystem Discovery Portal (https://data.niaid.nih.gov) provides a unified search interface for over 4 million data sets relevant to infectious and immune-mediated disease (IID) research. Integrating metadata from domain-specific and generalist repositories, the Portal enables researchers to identify and access data sets using user-friendly filters or advanced queries, without requiring technical expertise. The Portal supports discovery of a wide range of resources, including epidemiological, clinical, and multi-omic data sets and is designed to accommodate exploratory browsing and precise searches. The Portal provides filters, prebuilt queries, and data set collections to simplify the discovery process for users. The Portal additionally provides documentation and an API for programmatic access to harmonized metadata. By easing access barriers to important biomedical data sets, the NIAID Data Ecosystem Discovery Portal serves as an entry point for researchers working to understand, diagnose, or treat IID.

**IMPORTANCE:**

Valuable data sets are often overlooked because they are difficult to locate. The NIAID Data Ecosystem Discovery Portal fills this gap by providing a centralized, searchable interface that empowers users with varying levels of technical expertise to find and reuse data. By standardizing key metadata fields and harmonizing heterogeneous formats, the Portal improves data findability, accessibility, and reusability. This resource supports hypothesis generation, comparative analysis, and secondary use of public data by the IID research community, including those funded by NIAID. The Portal supports data sharing by standardizing metadata and linking to source repositories and maximizes the impact of public investment in research data by supporting scientific advancement via secondary use.

## INTRODUCTION

The biomedical research community has entered an era in which open science and data sharing are increasingly regarded as essential to accelerating discovery, improving reproducibility, and ensuring the integrity of publicly funded research. These expectations have been formalized by the U.S. National Institutes of Health (NIH) through its Data Management and Sharing Policy, which went into effect in 2023 and requires researchers to plan for and make their data publicly available whenever possible (1). Similar mandates from scientific journals, and funding agencies reflect a broader shift toward transparency and reuse in science (2).

While this emphasis on data sharing has led to a proliferation of public data sets (3), it has not eliminated the core challenges facing researchers who wish to reuse existing data. A critical and often overlooked barrier is the *discovery* of relevant data sets (4). Biomedical data are housed in hundreds of public repositories, both domain-specific and generalist, each with different metadata standards, access protocols, and search capabilities (5). As a result, finding data sets suitable for reuse often requires prior knowledge of where those data sets are hosted, what metadata they use, and how to navigate their interfaces. Even experienced data scientists frequently rely on *ad hoc* strategies, such as citation mining or keyword searching across multiple websites, to identify usable data sets (6).

These challenges are particularly acute in the field of **infectious and immune-mediated disease (IID)** research, which spans diverse experimental domains, from immunology and microbiology to clinical trials, imaging, and multi-omics. For example, a researcher studying host response to influenza may need to integrate transcriptomics data from GEO (7), immunophenotyping data from ImmPort (8), and viral sequences from GenBank (9). Yet each of these repositories uses its own metadata conventions, and none provides the means to search across them in a unified way. Inconsistent annotations for key properties such as species, pathogen, health condition, or assay type further impede cross-repository search even when the data are technically accessible.

Poorly formatted metadata remains a major obstacle, limiting the usability of otherwise available data. There is growing recognition that data set metadata is as crucial as the data sets themselves for ensuring that shared data can be found, interpreted, and reused. The FAIR data principles (Findable, Accessible, Interoperable, Reusable) have helped articulate this need by emphasizing not only data availability but also machine-readable, semantically rich, and standardized metadata (10). Efforts such as https://schema.org/ (11), Bioschemas (12), and the NIH Generalist Repository Ecosystem Initiative (GREI) (13) have begun to promote more consistent metadata practices. However, these initiatives rely heavily on repository participation and metadata provider compliance, something that is difficult to enforce and slow to propagate.

To address these problems, we developed the NIAID Data Ecosystem (NDE) Discovery Portal (https://data.niaid.nih.gov), a centralized interface that allows researchers to find data sets related to infectious and immune-mediated diseases regardless of where they are stored. Rather than attempting to centralize the data itself or enforce new metadata standards at the point of submission, our approach harmonizes metadata *post hoc* across multiple repositories using a custom schema based on existing standards. We then expose this harmonized metadata through a search engine tailored to the needs of IID researchers.

The rationale for this work is simple: shared data cannot fulfill its potential unless it can be readily found and reused. By focusing on post-hoc harmonization of repository metadata, a federated approach that respects the autonomy of participating repositories, and user-friendly search, the Discovery Portal enables researchers to identify relevant data sets for hypothesis generation, secondary analysis, and validation. In this resource report, we describe the development, design principles, and current capabilities of the NIAID Discovery Portal (RRID:SCR_027614), with particular attention to the value it provides to end users in the biomedical research community.

## RESULTS

The NDE Discovery Portal was developed to serve as a unified entry point for finding publicly available data sets relevant to infectious and immune-mediated disease (IID) research. Recognizing the diversity of experimental approaches and data types used in IID science, the Discovery Portal is designed to integrate metadata from a broad range of repositories spanning both domain-specific and generalist infrastructures. The result is a comprehensive, cross-repository index that allows researchers to identify relevant data sets without needing to know in advance which repository houses them or how that repository structures its metadata.

As of this writing, the Discovery Portal aggregates and harmonizes metadata from more than 4.3 million data sets across 42 repositories. These include prominent **domain-specific repositories** such as NCBI Sequence Read Archive (SRA) (14) for next-generation sequencing data, Gene Expression Omnibus (GEO) (7) for transcriptomics and functional genomics studies, ImmPort (8) for immunology-focused clinical and mechanistic studies, MassIVE (15) for mass spectrometry proteomics data sets, Qiita (16) for microbiome and metagenomic data, and COVID RADx Data Hub (17) for data from COVID-19 diagnostic and translational research. In addition, the Portal includes metadata from several **generalist repositories** supported by the NIH and other funders, including Figshare (18), Zenodo (19), Dryad (20), Harvard Dataverse (21), and Vivli (22). The home page includes a searchable table of all indexed repositories, annotated with data type, research domain, and access criteria (Table 1).

**TABLE 1:** Dataset repositories currently included in the NIAID Data Ecosystem

Name	Research Domain	Access
AccessClinicalData@NIAID	IID	Varied Access
AmoebaDB	IID	Registered Access
ClinEpiDB	IID	Varied Access
COVID RADx Data Hub	IID	Unknown Access
CryptoDB	IID	Registered Access
Data Discovery Engine	Generalist	Varied Access
Database of Genotypes and Phenotypes (dbGaP)	Generalist	Controlled Access
Dryad Digital Repository	Generalist	Open Access
Figshare	Generalist	Unknown Access
Flow Repository	Generalist	Unknown Access
FungiDB	IID	Registered Access
GiardiaDB	IID	Registered Access
Harvard Dataverse	Generalist	Varied Access
HostDB	IID	Registered Access
HuBMAP	Generalist	Varied Access
Human Cell Atlas	Generalist	Varied Access
ImmPort	IID	Registered Access
ImmuneSpace	IID	Registered Access
LINCS	Generalist	Unknown Access
MalariaGEN	IID	Varied Access
MassIVE	Generalist	Open Access
Mendeley Data	Generalist	Varied Access
MicrobiomeDB	IID	Open Access
MicrosporidiaDB	IID	Registered Access
NCBI BioProject	Generalist	Open Access
NCBI GEO	Generalist	Unknown Access
NCBI SRA	Generalist	Varied Access
NICHD Data and Specimen Hub (DASH)	Generalist	Controlled Access
OmicsDI	Generalist	Unknown Access
PiroplasmaDB	IID	Registered Access
PlasmoDB	IID	Registered Access
Qiita	IID	Registered
ReframeDB	IID	Controlled Access
The Network Data Exchange (NDEx)	Generalist	Open Access
ToxoDB	IID	Registered Access
TrichDB	IID	Registered Access
TriTrypDB	IID	Registered
VDJServer	IID	Open Access
VectorBase	IID	Registered Access
VEuPath Collections	IID	Registered Access
VEuPathDB	IID	Registered Access
Vivli	Generalist	Controlled Access
Zenodo	Generalist	Varied Access

This breadth allows researchers to query within a single interface for data sets across a wide spectrum of data modalities, from viral genomes to host transcriptomics, clinical trial data, and proteomic assays. For each integrated repository, metadata are harvested and mapped to a harmonized schema that aligns with FAIR principles, enabling consistent filtering and search across otherwise incompatible data sources.

**Fig 1:**
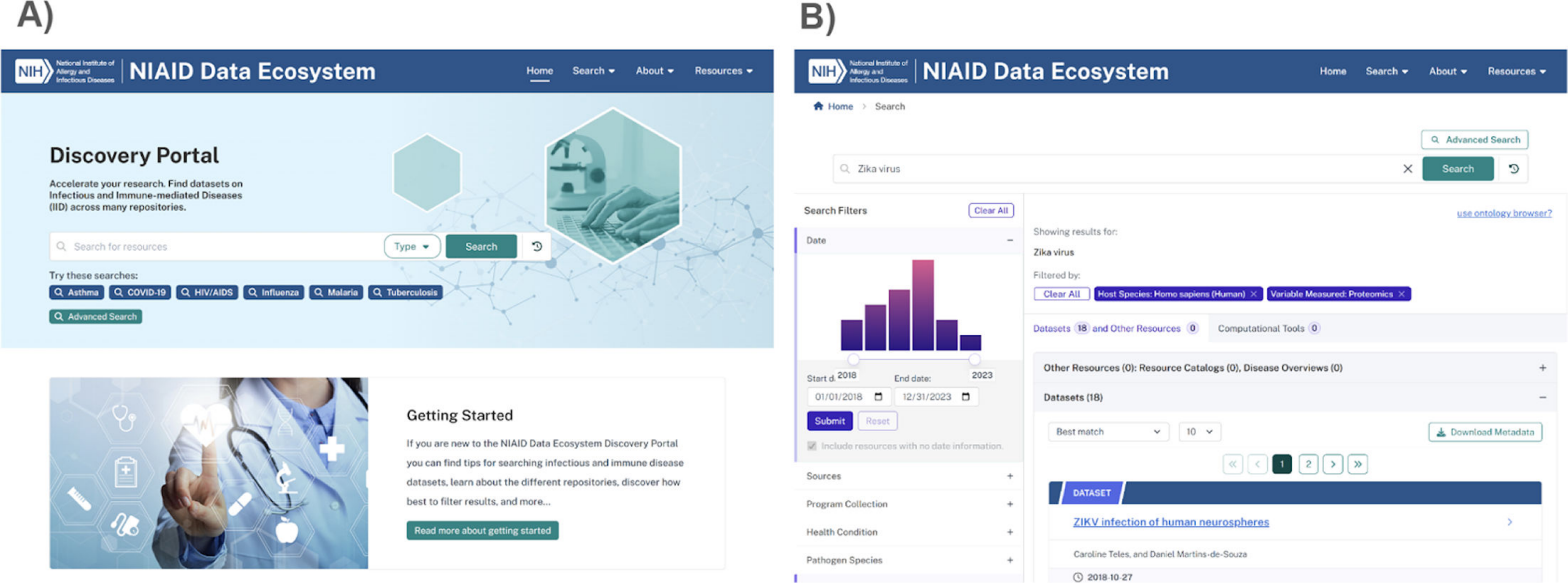
Overview of the NIAID data ecosystem interface and example search. (**A**) The landing page provides access to the Discovery Portal’s basic search interface. (**B**) Example search results for “Zika virus” data sets filtered by species: “Homo sapiens” and variableMeasured: “Proteomics,” demonstrating how users can refine results using structured metadata filters to support targeted data discovery.

Central to the utility of the Discovery Portal is a search interface that enables researchers to rapidly query the entire metadata index using free text or keyword-based input (Fig. 1A). This search is complemented by an array of intuitive, prebuilt filters that reflect metadata elements of particular interest to IID researchers. These filters include host species (e.g., *Homo sapiens*, *Mus musculus*), pathogen or infectious agent (e.g., *Mycobacterium tuberculosis*, *SARS-CoV-2*), health condition (e.g., asthma, HIV infection), and measurement technique (e.g., RNA-seq, flow cytometry).

These filters allow users to quickly refine results and identify data sets that are most relevant to their research needs. For example, a virologist can search for “Zika virus” and then restrict results to “Human” data sets measured via “Proteomics” in just a few clicks (Fig. 1B). The Portal also displays detailed metadata for each data set and provides direct links to the originating repository for data access. By leveraging the Portal’s harmonized metadata, researchers can refine their search, as well as perform downstream analyses across the data sets they identify.

For users with more complex information needs, the Portal also includes an advanced search interface that supports field-specific queries across nearly 50 metadata properties. This functionality is particularly useful for computational biologists, data managers, and tool developers who need to identify data sets with highly specific attributes, e.g., studies funded by a particular NIH grant, or data sets involving *Plasmodium falciparum* infection in nonhuman primates using ELISA assays. While the mechanics of query construction are hidden from users in the Basic Search interface, the Advanced Search option provides the flexibility to support compound filters, precise Boolean logic, and reproducible queries.

## DISCUSSION

The design of the NIAID Discovery Portal emphasized both the breadth of data set coverage and the accessibility of the search interface with the intent of providing a powerful tool for facilitating data reuse across the IID research landscape. This goal of promoting data reuse is shared across the NIH, with similar institute-specific initiatives in the NIDDK Central Repository (https://repository.niddk.nih.gov/), the Index of NCI Studies (https://studycatalog.cancer.gov/), and the NIMH Data Archive (https://nda.nih.gov/). While the NLM also offers a broadly-scoped effort in the NLM Data Catalog (https://datasetcatalog.nlm.nih.gov/), these domain-specific efforts allow for the development of more focused metadata schemas (e.g., the tracking of species and infectiousAgent in the the NIAID Discovery Portal) that are specifically relevant to certain research communities.

For example, a researcher studying immune response to influenza can enter “influenza” in the Portal’s search bar. This will return over 20,000 data sets. Then, the researcher can apply filters for species: “Homo sapiens” and measurementTechnique: “RNA-seq assay” to narrow the results to 215 data sets. This allows the researcher to explore multiple repositories at once without prior knowledge of individual databases, enhancing data discovery and enabling more efficient exploration across diverse sources and data types.

While the NDE Discovery Portal improves the findability and accessibility of IID data sets, several limitations remain that present opportunities for future development.

A primary challenge is the incompleteness and inconsistency of metadata across repositories. Many records lack key fields, such as species, infectiousAgent, or healthCondition, which are essential for effective filtering. Although metadata augmentation strategies have substantially improved coverage, they cannot fully compensate for missing or ambiguous source metadata. Furthermore, variations in term formatting (e.g., capitalization, punctuation, and hyphenation) can lead to duplicate or fragmented filter values, especially in free-text fields. Our standardization pipeline attempts to reconcile these differences, but consistent use of community identifiers would result in a more accurate result.

Another limitation involves semantic inconsistencies and limited context in generalist repositories. These platforms often include minimal required metadata, making it difficult to infer experimental detail or biological relevance that is necessary to enable domain-specific reuse. While additional metadata can sometimes be extracted from linked publications or supplementary materials, persistent identifiers (e.g., DOIs, PubMed IDs) are often not captured in a systematic and consistent manner.

From a technical standpoint, the Discovery Portal relies on a schema translation and indexing model that supports flexible integration but requires ongoing maintenance. Repository APIs, data formats, and metadata standards continue to evolve, necessitating regular updates to ingestion pipelines and harmonization logic. Maintaining a scalable, performant system while integrating new sources will remain a key operational challenge.

Looking forward, we plan to expand the scope and functionality of the Portal along several axes:

- **Incorporating additional resource types**, including computational tools, software pipelines, and models linked to data sets, to support reproducibility and reuse.
- **Improving entity resolution** through integration of cross-references (e.g., UMLS, MeSH, UniProt) and persistent identifiers, especially for diseases and organisms.
- **Supporting multilingual and lay-access metadata enhancements**, particularly for data sets relevant to global infectious disease surveillance and public health.
- **Exploring natural language and AI-assisted search interfaces**, including the integration of large language models (LLMs), to support more intuitive querying by non-specialist users.
- **Enhancing community engagement**, including support for user-contributed metadata curation, tagging, and annotation.

Ultimately, the continued value of the Discovery Portal will depend on its ability to evolve alongside the data practices and priorities of the IID research community. By identifying limitations and addressing them transparently, we aim to support a more FAIR, interconnected, and discoverable ecosystem of biomedical data.

## MATERIALS AND METHODS

### Metadata schema and mapping

To support consistent and effective search across diverse data repositories, the NDE Discovery Portal relies on a unified metadata schema that harmonizes descriptive information about data sets from disparate sources. The goal of this schema is to balance breadth and specificity, capturing a wide range of data set types across IID research while maintaining semantic consistency that supports meaningful filtering, indexing, and interoperability.

The NDE data set metadata schema was initially developed by the NIAID Systems Biology Data Dissemination Working Group (23). The approach is based on the Schema.org Data set class (11), a widely adopted standard in biomedical and general web search contexts. This foundation was extended with additional properties relevant to IID research, informed by user interviews, repository landscape analysis, and FAIR data principles.

The Dataset schema consists of a core set of **required, recommended, and optional fields**, including:

- **Required properties**
  - name (dataset title)
  - description
  - identifier (e.g., DOI or accession number)
  - url (link to the data source)
  - author—author or creator of the data
  - funding—grant or program that supported the data generation
  - measurementTechnique—methods used (e.g., RNA-seq, flow cytometry)
  - includedInDataCatalog—original repository
  - distribution—data download information
- **Recommended properties**
  - healthCondition—mapped to disease or clinical indication
  - infectiousAgent—pathogen or organism of study
  - species—host or model organism
  - variableMeasured—key variables or outputs
  - keywords—key terms helpful for search
  - doi—a digital object identifier for the data set itself (if available)
  - temporalCoverage, spatialCoverage—for epidemiological or longitudinal data
  - conditionsOfAccess, license, usageInfo—licensing or access terms
  - sdPublisher—the original publisher of the structured metadata if available
  - dateCreated, dateModified, datePublished—date information
  - citation, isBasedOn, citedBy—citation and reference information
- **Optional properties**:
  - hasPart, isPartOf, isRelatedTo, isSimilarTo, sameAs, isBasisFor—information on how the Data set relates to other types of CreativeWork
  - nctid—https://clinicaltrials.gov/ identifier if available
  - version—version information
  - abstract—used only when a source has both a summary/abstract and description information
  - isAccessibleForFree—boolean indicating whether or not there is a cost to access
  - sourceOrganization—a project, program, or organization for which the data were generated
  - Any other mappable https://schema.org/ Data set property that can be mapped to a property used by a resource

Wherever possible, metadata values are aligned with standard biomedical ontologies. For example, species and infectiousAgent values are mapped to NCBI Taxonomy IDs (24), healthCondition values are mapped Mondo Disease Ontology (MONDO) (25), Human Phenotype Ontology (HPO) (26), and Disease Ontology (DOID) (27)**,** topicCategoryvalues are mapped to EDAM (28), andfunding.identifiervalues are mapped to NIH grant numbers.

To facilitate metadata integration, each target repository is assessed for compatibility and relevance, with particular focus on repositories commonly cited in IID research or supported by NIAID funding. For each repository, a custom parser and transformation pipeline was implemented to extract, normalize, and map native metadata fields to the NDE schema. For instance:

- In **NCBI SRA**, elements such as study title, organism, and sequencing platform are extracted from XML records and mapped to name, species, and measurementTechnique, respectively.
- In **GEO**, metadata fields describing array or RNA-seq experiments are mapped to variableMeasured, measurementTechnique, and healthCondition, often leveraging accompanying PubMed references for context.
- In **ImmPort**, metadata about condition, species, and assay type can be directly mapped to healthCondition, species, and measurementTechnique.
- In **AccessClinicalData@NIAID**, clinical trial metadata, such as titles, identifiers, summaries, and conditions, are mapped to name, identifier, description, and healthCondition.
- In **VEuPathDB**, scientific context including organism, gene counts, and measurement types are mapped to species, variableMeasured, and measurementTechnique, respectively.
- For **generalist repositories** (e.g., Zenodo, Figshare), where metadata quality is more variable, the mapping pipeline prioritizes extraction of the most common standardized fields (e.g., name, description, doi) and infers additional properties when possible using linked resources such as citations.

Each repository-specific parser converts the native metadata into a JSON-LD (JavaScript Object Notation for Linked Data) document, a machine-readable format that standardizes metadata and conforms to the NDE schema. These documents are then indexed in Elasticsearch (29) and exposed via the Discovery Portal’s search interfaces and public API. Metadata is harvested every quarter, with the date of the latest update displayed on the Sources page. A basic search using the interface retrieves all records containing the search terms anywhere within the metadata record, including free-text fields such as keywords or descriptions. The ranking system prioritizes results with matches in structured fields, giving the most weight to the resource name.

To prevent redundancy, duplicate records are identified and harmonized wherever possible. Within repositories that assign new identifiers for each data set version, multiple entries for the same data set are consolidated by mapping all versions to a canonical record, with the canonical URL resolving to the latest version. For other repositories, duplicate records for the same data set are detected by matching primary identifiers. Unique metadata fields from each source are merged, prioritizing the primary source, while secondary-source fields are appended when unique. For the property includedInDataCatalog, all values are appended to preserve accurate provenance. To maintain integrity of original data sets, if we cannot verify that it is a true duplicate, we retain both copies.

In designing the NDE schema, we aligned our approach with broader community standards to ensure compatibility and interoperability. These include the Bioschemas initiative (12), which extends Schema.org to meet life science data needs, and GREI (13), which aims to improve metadata harmonization across NIH-supported generalist repositories. By drawing on these efforts, the NDE schema ensures alignment with emerging best practices and supports future interoperability with external search engines, FAIR data catalogs, and semantic web tools.

This schema-based harmonization strategy enables data sets from dozens of heterogeneous repositories to be searched, compared, and filtered in a coherent and consistent manner, without requiring repositories to adopt a new internal data model. Importantly, the use of open standards and publicly documented schema definitions also ensures that the Discovery Portal can support interoperability with external tools and data discovery platforms.

### Improving metadata completeness through ontology-based augmentation

Despite the adoption of a harmonized metadata schema, many records aggregated by the NIAID Discovery Portal lacked key metadata elements that are essential for meaningful filtering and data set discovery, particularly for fields like species, infectiousAgent, healthCondition, and funding. These gaps reflect the heterogeneity in metadata practices across repositories, particularly among generalist platforms and older records. To address this limitation and improve the user experience, we implemented a multi-pronged metadata augmentation strategy to enrich and standardize metadata values post-ingestion.

For structured fields such as species and infectiousAgent, we used Text2Term (30), a local ontology mapping tool, to match free-text values to standardized terms from the NCBI Taxonomy (24). We benchmarked our use of Text2Term against PubTator by pulling all PubTator-augmented organisms in our portal (167487) and mapping them using Text2Term. We found Text2Term successfully mapped 167,014 organisms (missing 473), and only differed from PubTator by 64 mappings, resulting in a difference of 537 (0.038%) in mapping outcomes. This process included extracting scientific, common, and alternative names as well as taxonomic identifiers, enabling consistent filter behavior and synonym resolution in the Discovery Portal interface. Since host species and pathogenic agents are both often provided under a single “organism” label in source repositories, we applied a taxonomy-based heuristic. This heuristic leveraged lineage information from the NCBI Taxonomy tree to classify organisms as potential hosts (e.g., vertebrates, arthropods, plants) or likely pathogens (e.g., bacteria, viruses, protozoa). Default assignments were manually reviewed for the most frequently occurring terms and iteratively refined based on user feedback. Occasional interchange between these fields may occur in rare cases due to variation in how a source stores organism information or incorrect mapping during heuristic assignment.

For the healthCondition field, we implemented a hierarchical ontology mapping workflow using the NCATS Translator Knowledge Provider (KP) APIs (31). These services allowed us to normalize disease-related terms to multiple interoperable ontologies, following a defined priority order: we first attempted to map to the Mondo Disease Ontology (MONDO) (25), then to the Human Phenotype Ontology (HPO) (26) if no MONDO match was found, followed by the Disease Ontology (DOID) (27), and finally the NCI Thesaurus (NCIT) (32).

This approach ensured broad coverage and alignment with downstream analysis tools and data standards. Each mapped term was stored along with its identifier, preferred label, and known synonyms.

### Leveraging publications to enrich metadata

When a data set had a linked primary PubMed-indexed publication, we used the citation as an external source of metadata to enhance the associated record. We extracted information from the publication, including diseases, organisms, and funding sources. For PubTator annotations (33) of disease and organism terms, we restricted inclusion to only terms that appeared in the data set’s name or description. PubTator annotations include the exact terms that were identified (and mapped) in an abstract and the same language is often used if the data set was provided by the same group. This ensured that only highly relevant terms were used for augmentation.

This citation-based strategy significantly improved metadata completeness, particularly for the healthCondition and funding fields. In many cases, metadata extracted from the publication was the only structured information available to identify the biological context of a data set.

### Text mining for unlinked records

For data sets lacking associated publications or standardized terms, we used the EXTRACT tool to identify biological concepts directly from free-text fields such as descriptionandname and then mapped the identified text using Text2Term. Having established Text2Term as performing on par with PubTator, we used Text2Term as a means of improving the mapping of the EXTRACT-identified terms. While this method is inherently noisier, it provides valuable metadata for records that would otherwise be unannotated. To improve the quality of the EXTRACT-T2T results, we reviewed a sample of ~1,200 extracted species for uncommon values and manually checked the uncommon values for accuracy. Based on this approach, we created an initial list of false positives for removal and established a protocol for reporting (including GH issue templates/forms), reviewing and removing/correcting incorrectly inferred terms.

**Fig 2:**
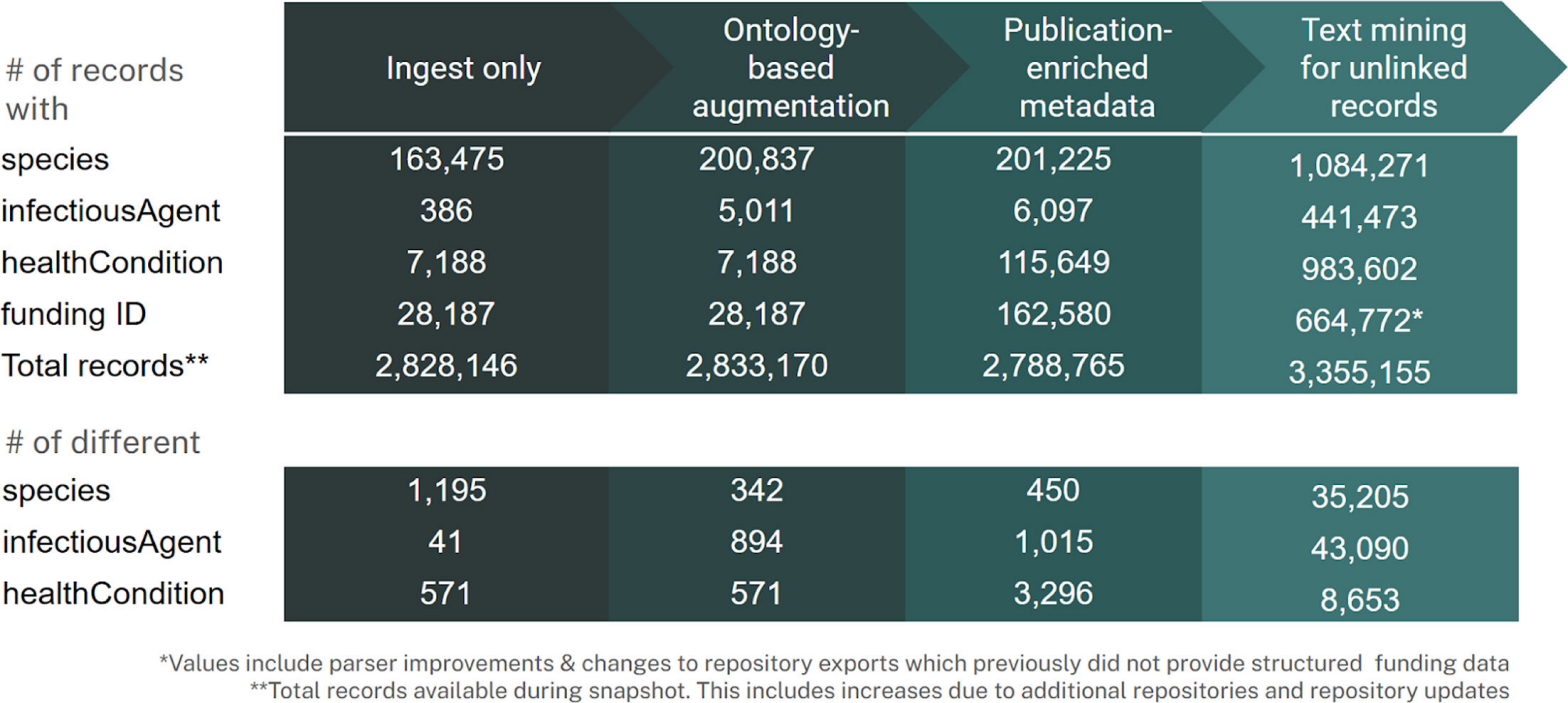
Overview of metadata improvements.

Based on the metadata augmentation pipelines above, we were able to substantially increase the completeness in our metadata catalog (Fig. 2).

We used PubTator (33) for free-text augmentation when PubMed IDs were available and EXTRACT when they were not. As both tools were evaluated by the BioCreative challenge (34), we used these benchmarks and focused our internal quality control efforts on iteratively removing terms that EXTRACT frequently mislabels. These corrections are maintained in a public corrections repository. For structured field standardization, we use the ontology mapping tool, Text2Term (30) for organisms and a hierarchical ontology mapping workflow of NCATS Translator Knowledge Provider (KP) APIs (31) for health conditions.

### Augmentation of topic categories

To improve domain-level filtering and support topic-based navigation, we added the topicCategory field to each record using the EDAM Topics ontology (28). An initial set of ~380 records across all repositories was manually annotated and used as a benchmark to evaluate classification performance by a large language model (GPT-4.5) (35). The model’s predictions were scored against human raters using an adjusted agreement metric that accounted for EDAM’s hierarchical structure. Briefly, two topics were considered a match if they were in the same branch, but penalties were applied based on closeness to root (i.e., being overly broad), and number of steps between terms (i.e., term distance). Based on its consistent performance, the model was used to annotate over 1.8 million records with EDAM topic classifications.

**Fig 3:**
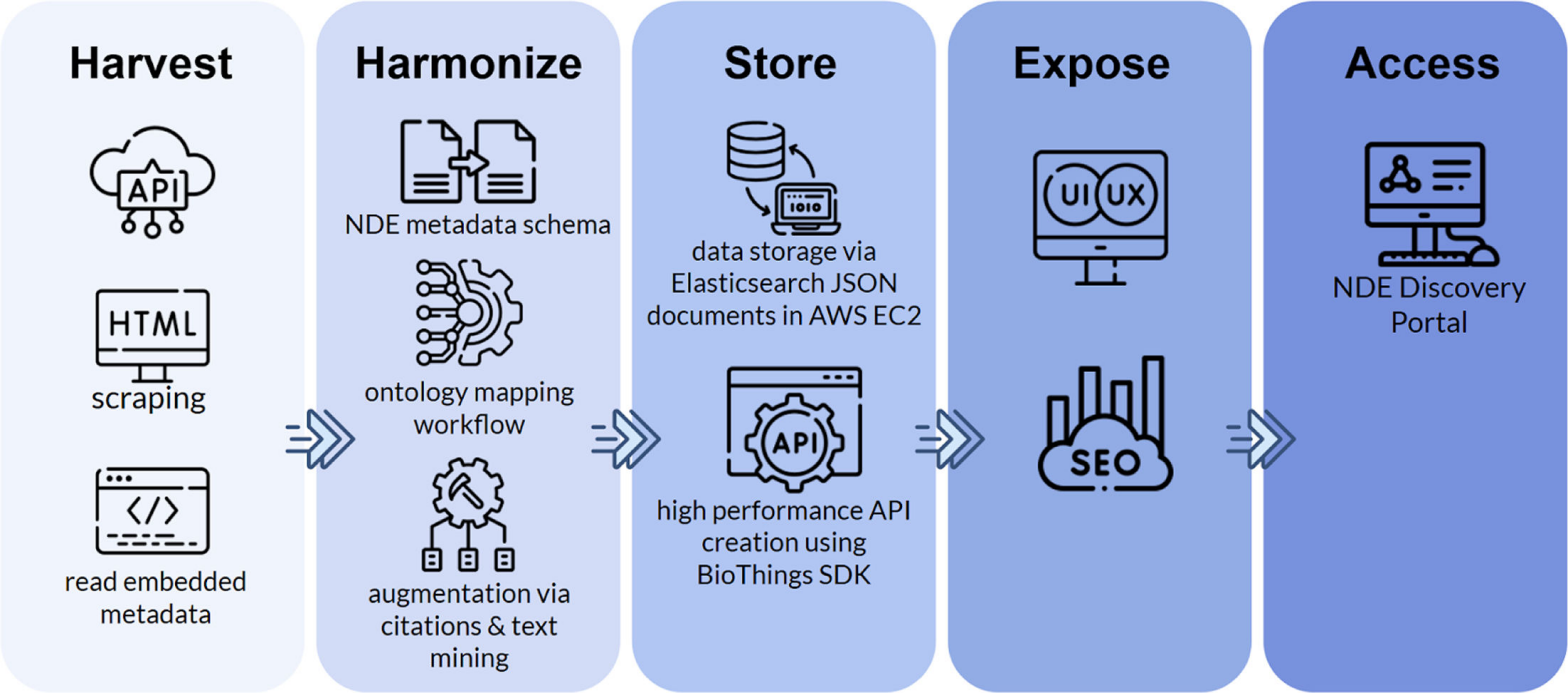
Overview of the NIAID data ecosystem metadata integration pipeline. Metadata is collected from repositories using APIs, HTML scraping, or by reading embedded metadata. Harvested metadata is converted to the NDE schema and standardized using ontology mapping workflows. Additional enrichment is performed through citation mining and text-based augmentation to improve standardization. Harmonized metadata is stored as JSON documents in Elasticsearch, hosted on AWS EC2. A high-performance API was built using BioThings SDK to enable scalable data access. The indexed data are exposed through a search interface designed for discoverability, usability (UI/UX), and search engine optimization (SEO). Users access the metadata through the NDE Discovery Portal.

By augmenting and standardizing metadata across key fields, we significantly improved the completeness, consistency, and usability of records in the Discovery Portal. These efforts not only enhance the precision and relevance of search results but also increase the visibility of data sets that might otherwise remain hidden due to poor metadata quality. Together with schema harmonization, metadata augmentation is a critical component of our strategy to make IID-related data sets more FAIR and actionable for the research community (Fig. 3).

### Outreach and usage metrics

To promote awareness and adoption of the NIAID Discovery Portal, we implemented a multifaceted outreach strategy targeting the IID research community. This included presentations at NIH workshops and scientific conferences, targeted email campaigns to NIAID-funded investigators, social media engagement, and content development for blogs and data science forums. In parallel, we optimized the Portal for discoverability via search engine optimization (SEO) strategies (36), including enhanced metadata tagging, improved mobile performance, and backlink generation from trusted scientific and institutional websites.

These efforts led to a steady increase in Portal engagement. Since its launch, the Discovery Portal has attracted over 100,000 unique users, with more than 180,000 pageviews and growing. Usage analytics show that both the basic search and advanced search functionalities are regularly used (though basic search is used approximately 30 times more often than advanced search), with a notable proportion of sessions involving metadata filtering by species, pathogen, or health condition. Usage analytics also show that 90% of sessions reach and end on a data set page. Peaks in user activity have consistently corresponded with outreach events, underscoring the importance of sustained community engagement.

The Portal currently receives over 10,000 monthly users, reflecting its growing role as a central resource for data discovery in the IID research community. In addition, we have also collaborated with several IID domain specific research programs, such as the Systems Biology Consortium for Infectious Diseases (37), Centers for Research in Emerging Infectious Diseases (CREID) Network (38), and Research and Development of Vaccines and Monoclonal Antibodies for Pandemic Preparedness (REVAMPP) (39), to make their data sets more discoverable and accessible by researchers.

## Data Availability

Portal: https://data.niaid.nih.gov API: https://api.data.niaid.nih.gov Metadata schema: https://discovery.biothings.io/ns/nde Source code: Portal source code: https://github.com/NIAID-Data-Ecosystem/nde-portal API source code: https://github.com/NIAID-Data-Ecosystem/nde-discovery-api Plugins source code: https://github.com/NIAID-Data-Ecosystem/nde-crawlers.
